# Supplementary material for: Phenotypic ranking experiments in identifying breeding objective traits of smallholder farmers in northwestern Ethiopia
Source: PLoS One. 2021 Mar 25;16(3):e0248779. doi: 10.1371/journal.pone.0248779 (PMC7993801; doi:10.1371/journal.pone.0248779)
Supplement: S2 File — (DOCX) [file pone.0248779.s002.docx]

**R script for frequency and proportion of breeding Arab doe traits data analysis (own-flock ranking)**

library(readxl)

Ranking <- read_excel("D:/Files in D/Ranking experiments/Ranking.xlsx",

sheet = "Arab doe (own-flock attributes)"

View(Ranking)

library(gmodels)

CrossTable(Ranking$Body size)

CrossTable(Ranking$coat color)

CrossTable(Ranking$kid growth)

CrossTable(Ranking$mothering ability)

CrossTable(Ranking$twinning rate)

CrossTable(Ranking$kidding interval)

CrossTable(Ranking$kid size)

CrossTable(Ranking$body condition)

CrossTable(Ranking$drought tolerance)

CrossTable(Ranking$body conformation)

CrossTable(Ranking$body length)

CrossTable(Ranking$temparament)

CrossTable(Ranking$sex of kid)

CrossTable(Ranking$age at puberty)

CrossTable(Ranking$pedigree)

CrossTable(Ranking$foraging ability)

CrossTable(Ranking$body width)

savehistory("D:/Files in D/Ranking experiments/Homosha (Doe-own flock)...freq. and percentage")

**R script for Arab doe measurements data analysis (own-flock ranking)**

library(readxl)

Ranking <- read_excel("D:/Files in D/Ranking experiments/Ranking.xlsx",

sheet = "Arab doe (own-flock measuremen)")

View(Ranking)

mean(Ranking$`Dentition 1st`)

sd(Ranking$`Dentition 1st`)/sqrt(length(Ranking$`Dentition 1st`))

mean(Ranking$`Dentition 2nd`)

sd(Ranking$`Dentition 2nd`)/sqrt(length(Ranking$`Dentition 2nd`))

mean(Ranking$`Dentition 3rd`)

sd(Ranking$`Dentition 3rd`)/sqrt(length(Ranking$`Dentition 3rd`))

mean(Ranking$`Dentition worst`)

sd(Ranking$`Dentition worst`)/sqrt(length(Ranking$`Dentition worst`))

mean(Ranking$`BW 1st`)

sd(Ranking$`BW 1st`)/sqrt(length(Ranking$`BW 1st`))

mean(Ranking$`BW 2nd`)

sd(Ranking$`BW 2nd`)/sqrt(length(Ranking$`BW 2nd`))

mean(Ranking$`BW 3rd`)

sd(Ranking$`BW 3rd`)/sqrt(length(Ranking$`BW 3rd`))

mean(Ranking$`BW worst`)

sd(Ranking$`BW worst`)/sqrt(length(Ranking$`BW worst`))

mean(Ranking$`NK 1st`)

sd(Ranking$`NK 1st`)/sqrt(length(Ranking$`NK 1st`))

mean(Ranking$`NK 2nd`)

sd(Ranking$`NK 2nd`)/sqrt(length(Ranking$`NK 2nd`))

mean(Ranking$`NK 3rd`)

sd(Ranking$`NK 3rd`)/sqrt(length(Ranking$`NK 3rd`))

mean(Ranking$`NK worst`)

sd(Ranking$`NK worst`)/sqrt(length(Ranking$`NK worst`))

mean(Ranking$`Twining 1st`)

sd(Ranking$`Twining 1st`)/sqrt(length(Ranking$`Twining 1st`))

mean(Ranking$`Twinning 2nd`)

sd(Ranking$`Twinning 2nd`)/sqrt(length(Ranking$`Twinning 2nd`))

mean(Ranking$`Twinning 3rd`)

sd(Ranking$`Twinning 3rd`)/sqrt(length(Ranking$`Twinning 3rd`))

mean(Ranking$`Twinning worst`)

sd(Ranking$`Twinning worst`)/sqrt(length(Ranking$`Twinning worst`))

mean(Ranking$`NB 1st`)

sd(Ranking$`NB 1st`)/sqrt(length(Ranking$`NB 1st`))

mean(Ranking$`NB 2nd`)

sd(Ranking$`NB 2nd`)/sqrt(length(Ranking$`NB 2nd`))

mean(Ranking$`NB 3rd`)

sd(Ranking$`NB 3rd`)/sqrt(length(Ranking$`NB 3rd`))

mean(Ranking$`NB worst`)

sd(Ranking$`NB worst`)/sqrt(length(Ranking$`NB worst`))

mean(Ranking$`NW 1st`)

sd(Ranking$`NW 1st`)/sqrt(length(Ranking$`NW 1st`))

mean(Ranking$`NW 2nd`)

sd(Ranking$`NW 2nd`)/sqrt(length(Ranking$`NW 2nd`))

mean(Ranking$`NW 3rd`)

sd(Ranking$`NW 3rd`)/sqrt(length(Ranking$`NW 3rd`))

mean(Ranking$`NW worst`)

sd(Ranking$`NW worst`)/sqrt(length(Ranking$`NW worst`))

**R script for frequency and proportion of breeding Arab doe traits data analysis (group-animal ranking)**

View(Ranking)

library(readxl)

Ranking <- read_excel("D:/Files in D/Ranking experiments/Ranking.xlsx",

sheet = "Hom (Doe group-animal attribute")

library(gmodels)

CrossTable(Ranking$`body conformation`)

CrossTable(Ranking$`body size`)

CrossTable(Ranking$`coat color`)

CrossTable(Ranking$`color pattern`)

CrossTable(Ranking$`body width`)

CrossTable(Ranking$`body condition`)

CrossTable(Ranking$age)

CrossTable(Ranking$age)

CrossTable(Ranking$`horn length`)

CrossTable(Ranking$`body length`)

CrossTable(Ranking$`beauty/appearance`)

CrossTable(Ranking$`ear size`)

CrossTable(Ranking$height)

CrossTable(Ranking$`kidding interval`)

CrossTable(Ranking$`twinning rate`)

CrossTable(Ranking$`mothering ability`)

**R script for frequency and proportion of breeding Arab buck traits data analysis (group-animal ranking)**

library(readxl)

Ranking <- read_excel("D:/Files in D/Ranking experiments/Ranking.xlsx",

sheet = "Arab buck (group-animal attrib")

View(Ranking)

CrossTable(Ranking$`coat color`)

CrossTable(Ranking$`body size`)

CrossTable(Ranking$`body width`)

CrossTable(Ranking$`body conformation`)

CrossTable(Ranking$`body length`)

CrossTable(Ranking$`body condition`)

CrossTable(Ranking$age)

CrossTable(Ranking$libido)

CrossTable(Ranking$`horn shape`)

CrossTable(Ranking$appearance)

CrossTable(Ranking$`horn orientation`)

CrossTable(Ranking$`ear size`)

CrossTable(Ranking$`horn size`)

CrossTable(Ranking$temparament)

CrossTable(Ranking$height)

CrossTable(Ranking$`fast growth`)

CrossTable(Ranking$`color pattern`)

CrossTable(Ranking$`heat tolerance`)

**R script for frequency and proportion of breeding Oromo doe traits data analysis (own-flock ranking)**

library(readxl)

Ranking <- read_excel("D:/Files in D/Ranking experiments/Ranking.xlsx",

sheet = "Oromo doe (own-flock attribute)")

View(Ranking)

library(gmodels)

CrossTable(Ranking$`Body size`)

CrossTable(Ranking$`coat color`)

CrossTable(Ranking$`kid growth`)

CrossTable(Ranking$`kidding interval`)

CrossTable(Ranking$`twinning rate`)

CrossTable(Ranking$`mothering ability`)

CrossTable(Ranking$`kid size`)

CrossTable(Ranking$`body condition`)

CrossTable(Ranking$`drought tolerance`)

CrossTable(Ranking$`body conformation`)

CrossTable(Ranking$`body length`)

CrossTable(Ranking$temparament)

CrossTable(Ranking$`sex of kid`)

CrossTable(Ranking$`age at puberty`)

CrossTable(Ranking$pedigree)

CrossTable(Ranking$`foraging ability`)

CrossTable(Ranking$`body width`)

**R script for Oromo doe measurements data analysis (own-flock ranking)**

library(readxl)

Ranking <- read_excel("D:/Files in D/Ranking experiments/Ranking.xlsx",

sheet = "Oromo doe (own-flock measureme)")

View(Ranking)

mean(Ranking$`Dentition 1st`)

sd(Ranking$`Dentition 1st`)/sqrt(length(Ranking$`Dentition 1st`))

mean(Ranking$`Dentition 2nd`)

sd(Ranking$`Dentition 2nd`)/sqrt(length(Ranking$`Dentition 2nd`))

mean(Ranking$`Dentition 3rd`)

sd(Ranking$`Dentition 3rd`)/sqrt(length(Ranking$`Dentition 3rd`))

mean(Ranking$`Dentition worst`)

sd(Ranking$`Dentition worst`)/sqrt(length(Ranking$`Dentition worst`))

mean(Ranking$`BW 1st`)

sd(Ranking$`BW 1st`)/sqrt(length(Ranking$`BW 1st`))

mean(Ranking$`BW 2nd`)

sd(Ranking$`BW 2nd`)/sqrt(length(Ranking$`BW 2nd`))

mean(Ranking$`BW 3rd`)

sd(Ranking$`BW 3rd`)/sqrt(length(Ranking$`BW 3rd`))

mean(Ranking$`BW worst`)

sd(Ranking$`BW worst`)/sqrt(length(Ranking$`BW worst`))

mean(Ranking$`NK 1st`)

sd(Ranking$`NK 1st`)/sqrt(length(Ranking$`NK 1st`))

mean(Ranking$`NK 2nd`)

sd(Ranking$`NK 2nd`)/sqrt(length(Ranking$`NK 2nd`))

mean(Ranking$`NK 3rd`)

sd(Ranking$`NK 3rd`)/sqrt(length(Ranking$`NK 3rd`))

mean(Ranking$`NK worst`)

sd(Ranking$`NK worst`)/sqrt(length(Ranking$`NK worst`))

mean(Ranking$`Twining 1st`)

sd(Ranking$`Twining 1st`)/sqrt(length(Ranking$`Twining 1st`))

mean(Ranking$`Twinning 2nd`)

sd(Ranking$`Twinning 2nd`)/sqrt(length(Ranking$`Twinning 2nd`))

mean(Ranking$`Twinning 3rd`)

sd(Ranking$`Twinning 3rd`)/sqrt(length(Ranking$`Twinning 3rd`))

mean(Ranking$`Twinning worst`)

sd(Ranking$`Twinning worst`)/sqrt(length(Ranking$`Twinning worst`))

mean(Ranking$`NB 1st`)

sd(Ranking$`NB 1st`)/sqrt(length(Ranking$`NB 1st`))

mean(Ranking$`NB 2nd`)

sd(Ranking$`NB 2nd`)/sqrt(length(Ranking$`NB 2nd`))

mean(Ranking$`NB 3rd`)

sd(Ranking$`NB 3rd`)/sqrt(length(Ranking$`NB 3rd`))

mean(Ranking$`NB worst`)

sd(Ranking$`NB worst`)/sqrt(length(Ranking$`NB worst`))

mean(Ranking$`NW 1st`)

sd(Ranking$`NW 1st`)/sqrt(length(Ranking$`NW 1st`))

mean(Ranking$`NW 2nd`)

sd(Ranking$`NW 2nd`)/sqrt(length(Ranking$`NW 2nd`))

mean(Ranking$`NW 3rd`)

sd(Ranking$`NW 3rd`)/sqrt(length(Ranking$`NW 3rd`))

mean(Ranking$`NW worst`)

sd(Ranking$`NW worst`)/sqrt(length(Ranking$`NW worst`))

**R script for frequency and proportion of breeding Oromo buck traits data analysis (group-animal ranking)**

library(readxl)

Ranking <- read_excel("D:/Files in D/Ranking experiments/Ranking.xlsx",

sheet = "Oromo doe(group-animal attrib")

View(Ranking)

CrossTable(Ranking$`body conformation`)

CrossTable(Ranking$`body size`)

CrossTable(Ranking$`coat color`)

CrossTable(Ranking$`color pattern`)

CrossTable(Ranking$`body width`)

CrossTable(Ranking$`body condition`)

CrossTable(Ranking$age)

CrossTable(Ranking$`horn length`)

CrossTable(Ranking$`body length`)

CrossTable(Ranking$`beauty/appearance`)

CrossTable(Ranking$`ear size`)

CrossTable(Ranking$height)

CrossTable(Ranking$`mothering ability`)

CrossTable(Ranking$`twinning ability`)

CrossTable(Ranking$`kidding interval`)

**R script for frequency and proportion of breeding Oromo buck traits data analysis (group-animal ranking)**

library(readxl)

Ranking <- read_excel("D:/Files in D/Ranking experiments/Ranking.xlsx",

sheet = "Oromo buck (group-animal attri")

View(Ranking)

CrossTable(Ranking$`coat color`)

CrossTable(Ranking$`body size`)

CrossTable(Ranking$`body width`)

CrossTable(Ranking$`body conformation`)

CrossTable(Ranking$`body length`)

CrossTable(Ranking$`body condition`)

CrossTable(Ranking$age)

CrossTable(Ranking$libido)

CrossTable(Ranking$`horn shape`)

CrossTable(Ranking$appearance)

CrossTable(Ranking$`horn orientation`)

CrossTable(Ranking$`horn size`)

CrossTable(Ranking$temparament)

CrossTable(Ranking$height)

CrossTable(Ranking$`fast growth`)

CrossTable(Ranking$`color pattern`)

CrossTable(Ranking$`heat tolerance`)
