# Supplementary material for: Phenotypic ranking experiments in identifying breeding objective traits of smallholder farmers in northwestern Ethiopia
Source: PLoS One. 2021 Mar 25;16(3):e0248779. doi: 10.1371/journal.pone.0248779 (PMC7993801; doi:10.1371/journal.pone.0248779)
Supplement: S3 File — (DOCX) [file pone.0248779.s003.docx]

1. **Recording format for phenotypic ranking of does by their owners**

Owner’s name__________________________ Location______________ Flock size_______ Number of breeding does________

| No | Traits | 1^st^ best | 2^nd^ best | 3^rd^ best | Worst | Remark |
| --- | --- | --- | --- | --- | --- | --- |
| 1 | Body weight |  |  |  |  |  |
| 2 | Body length |  |  |  |  |  |
| 3 | Wither height |  |  |  |  |  |
| 4 | Chest girth |  |  |  |  |  |
| 5 | Age/dentition |  |  |  |  |  |
| 6 | Number of kidding |  |  |  |  |  |
| 7 | Twining |  |  |  |  |  |
| 8 | No. kids born |  |  |  |  |  |
| 9 | No. kids weaned |  |  |  |  |  |
| Reasons for ranking |  |  |  |  |  |  |

**2. Recording format for bucks life history (as recalled by owner)**

Owner’s name__________________________ Location______________

| No | Traits | Description |
| --- | --- | --- |
| 1 | Age |  |
| 2 | Birth type |  |
| 3 | Live weight |  |
| 4 | Libido |  |
| 5 | Temperament |  |

**3. Recording format for ranking before getting life history information on individual doe in group-animal ranking**

| Pen 1 | | Pen 2 | | Pen 3 | | Pen 4 | |
| --- | --- | --- | --- | --- | --- | --- | --- |
| ID | Rank | ID | Rank | ID | Rank | ID | Rank |
|  |  |  |  |  |  |  |  |
|  |  |  |  |  |  |  |  |
|  |  |  |  |  |  |  |  |

Reasons ___________________________________________________________________________________________________________________________________________________________________________________________________________________________________________________________________________________________________________________________________________________________________________________________________________________________________________________________________________________________________________________________________________________________________________________________________________________

**4. Recording format for ranking after getting information on individual buck in group ranking**

| Pen 1 | | Pen 2 | | Pen 3 | | Pen 4 | |
| --- | --- | --- | --- | --- | --- | --- | --- |
| ID | Rank | ID | Rank | ID | Rank | ID | Rank |
|  |  |  |  |  |  |  |  |
|  |  |  |  |  |  |  |  |
|  |  |  |  |  |  |  |  |

Reasons ___________________________________________________________________________________________________________________________________________________________________________________________________________________________________________________________________________________________________________________________________________________________________________________________________________________________________________________________________________________________________________________________________________________________________________________________________________________
