# Supplementary material for: Phenotypic ranking experiments in identifying breeding objective traits of smallholder farmers in northwestern Ethiopia
Source: PLoS One. 2021 Mar 25;16(3):e0248779. doi: 10.1371/journal.pone.0248779 (PMC7993801; doi:10.1371/journal.pone.0248779)
Supplement: Attachment — Submitted filename: Response to Reviewers.docx [file pone.0248779.s004.docx]

### Responses to the Editor’s and Reviewers' Comments

Dear editor and reviewers, we would like to thank you for giving us a chance to revise our manuscript. We are also very thankful for your thoughtful and thorough review of our manuscript. The comments are encouraging and you appear to share our judgment that the study and its results are important. Each comment has been carefully considered point by point and responded accordingly. Please see below, in blue, our response to your comments. All line numbers given in the authors’ response refer to the revised manuscript and the revision can be seen as track changes in the manuscript.

**RESPONSE TO EDITOR’S COMMENTS**

1. Please ensure that your manuscript meets PLOS ONE's style requirements, including those for file naming. The PLOS ONE style templates can be found at

https://journals.plos.org/plosone/s/file?id=wjVg/PLOSOne_formatting_sample_main_body.pdf and

https://journals.plos.org/plosone/s/file?id=ba62/PLOSOne_formatting_sample_title_authors_affiliations.pdf

**Authors’ response:** Dear Editor, thank you so much for providing us the link. We have read it carefully and revised our manuscript according to the guideline.

2. We suggest you thoroughly copyedit your manuscript for language usage, spelling, and grammar. If you do not know anyone who can help you do this, you may wish to consider employing a professional scientific editing service.

**Authors’ response:** Dear, thanks for your concern. Indeed, we have tried all our best to improve the language usage, spelling, and grammar and make the manuscript clear to the readers.

3. We note that you have indicated that data from this study are available upon request. PLOS only allows data to be available upon request if there are legal or ethical restrictions on sharing data publicly. For more information on unacceptable data access restrictions, please see http://journals.plos.org/plosone/s/data-availability#loc-unacceptable-data-access-restrictions.

In your revised cover letter, please address the following prompts:

a) If there are ethical or legal restrictions on sharing a de-identified data set, please explain them in detail (e.g., data contain potentially sensitive information, data are owned by a third-party organization, etc.) and who has imposed them (e.g., an ethics committee). Please also provide contact information for a data access committee, ethics committee, or other institutional body to which data requests may be sent.

b) If there are no restrictions, please upload the minimal anonymized data set necessary to replicate your study findings as either Supporting Information files or to a stable, public repository and provide us with the relevant URLs, DOIs, or accession numbers. For a list of acceptable repositories, please see http://journals.plos.org/plosone/s/data-availability#loc-recommended-repositories.

We will update your Data Availability statement on your behalf to reflect the information you provide.

**Authors’ response:** Dear Editor, thanks for your question. All relevant data are within the manuscript and its supporting information files.

4. Your ethics statement should only appear in the Methods section of your manuscript. If your ethics statement is written in any section besides the Methods, please move it to the Methods section and delete it from any other section. Please ensure that your ethics statement is included in your manuscript, as the ethics statement entered into the online submission form will not be published alongside your manuscript.

**Authors’ response:** We thank you for your comment; we moved the ethics statement to the methods section of our manuscript.

5. Thank you for stating the following in the Funding Section of your manuscript:

"The financial support for this study was provided by Biotechnology Research Institute (BRI) of

Bahir Dar University, Ethiopia."

We note that you have provided funding information that is not currently declared in your Funding Statement. However, funding information should not appear in the Acknowledgments section or other areas of your manuscript. We will only publish funding information present in the Funding Statement section of the online submission form.

Please remove any funding-related text from the manuscript and let us know how you would like to update your Funding Statement. Currently, your Funding Statement reads as follows:

"The authors received no specific funding for this research".

Please include your amended statements within your cover letter; we will change the online submission form on your behalf.

**Authors’ response:** Dear Editor, thanks for the comment. We removed the funding statement that appeared in the acknowledgement section of our manuscript. On the other hand, we included our amended statements within our cover letter.

**RESPONSE TO REVIEWERS’ COMMENTS**

**Reviewer’s comment:**

Reviewer #1: Umer et al. investigated goat phenotype ranking schemes for identification breeding objective traits of smallholder farmers in Northwestern Ethiopia. For this, the authors looked into Own-flock ranking scheme in which the farmers ranked does based on age, number of kidding, twinning ability, number of kids born per kidding and number of kids weaned and Group-animal ranking scheme in which breeding does and bucks were randomly selected and phenotype information was gathered and then the farmers were asked to rank the animals with and without phenotype information. Accordingly, the author indicated that body size, mothering ability, twinning rate, kidding interval and temperament are prioritized traits while in group-animal ranking scheme, traits including coat color, body size and body conformation were found to be the priority of farmers. All in all, this study is relevant and a step forward to identify relevant to establish goat breeding objectives that could fit to smallholder farmers in Northwestern Ethiopia. Nevertheless, before this article is accepted for publication, the following issues should be addressed.

1. Statistical testing in the majority of results provided in tables are missing

**Authors’ Response:** Dear reviewer, thank you for your comment. We believe that statistical testing is needed and we included it in Table 2. The rest of the results in Tables 1, 3, 4 and 5 are qualitative data which, we believe, do not need any significance tests.

2. Tables 3, 4 and 5 are all described as Table 3-- please correct!

**Authors’ Response:** We appreciate your comment. Sorry for the mistake we made. Now, we correct it and you may find the correction in the revised manuscript.

3. In table 2 - Please indicate possible significant differences between 1 and 2, 1 and 3 and /or 2 and 3 rakings using letters or any convenient symbols.

**Authors’ Response:** Dear reviewer, we appreciate you. Based on your suggestion, we indicate the possible significant differences between the 1^st^ and 2^nd^, 1^st^ and 3^rd^, 1^st^ and inferior, 2nd and 3^rd^, 2^nd^ and inferior, and 3^rd^ and inferior rakings using letters.

4. Please describe if there is any goat trait preference differences between the Oromo and Arab smallholder goat farmers.

**Authors’ Response:** Dear reviewer, thank you so much for your comment. The goat trait preference difference between the Arab and Oromo goat keepers is not the objective of the present study. This issue is addressed in a previous study conducted by the same authors of the current study in 2019. For more information, you can refer an article entitled with “Production systems and breeding practices of Arab and Oromo goat keepers in northwestern Ethiopia: implications for community-based breeding programs” TROPICAL ANIMAL HEALTH AND PRODUCTION. 52, 1467–1478.

5. In the conclusion part of this manuscript, the authors indicated that in the own-flock ranking scheme farmers were interested in productive, reproductive and behavioral traits whereas in the group-animal ranking scheme, farmers were interested in coat color, body size and body conformation. It will be interesting if the authors could address this controversy.

**Authors’ Response:** Yes. In the conclusion part of our manuscript, we indicated that goat keepers in the own-flock ranking experiment were interested in productive, reproductive and behavioral traits whereas in the group-animal ranking experiment, they were interested in coat color, body size and body conformation. This could be due to the reason that keepers in the own-flock ranking experiment know the animals very well since they are the owners of the goats whereas in the group-animal ranking experiment, goat keepers rank animals brought from another area, hence they did not know them. Thus, this controversy was happened due to the knowledge of the goat keepers that they had on the ranked animals (ranking either their own goats or unknown goats). This issue is addressed well in the methods section of our manuscript (i.e., own-flock ranking experiment and group animal ranking experiment). As a result of this, we believe that it would be redundant if we address this same issue in the conclusion part.

Reviewer #2: Authors carried out own-flock and group-animal ranking experiments on two goat breeds, the Arab and Oromo breeds, in northwestern Ethiopia. More than 15 productive, reproductive and behavioral traits were used to rank the animals according to the choice of the farmers. This is a very great effort in designing a breeding program at a community level. The paper is written very well. Authors need to state the result section as “result and discussion”. Moreover, supplementing a representative picture of the goats from each breed, with respect to the traits mentioned in Table 2 would enhance the manuscript.

**Authors’ Response:** We are grateful for your comment. Now, we state the result section as “Result and discussion”. Furthermore, we depicted the pictures of the goat populations in Figure 1.

Best Regards,

Oumer Sheriff, on behalf of the authors
